# Editorial: The Functional Anatomy of the Reticular Formation

**DOI:** 10.3389/fnana.2019.00055

**Published:** 2019-05-29

**Authors:** Ugo Faraguna, Michela Ferrucci, Filippo S. Giorgi, Francesco Fornai

**Affiliations:** ^1^Department of Translational Research and New Technologies in Medicine and Surgery, University of Pisa, Pisa, Italy; ^2^Section of Neurology, Department of Clinical and Experimental Medicine, Pisa University Hospital, University of Pisa, Pisa, Italy; ^3^I.R.C.C.S. I.N.M. Neuromed, Pozzilli, Italy

**Keywords:** sleep-wake cycle, arousal, emotional brainstem, locus coereleus, pain, iso-dendritic neurons, catecholamine, drug addiction

The brainstem reticular formation (RF) represents the archaic core of those pathways connecting the spinal cord and the encephalon. It subserves autonomic, motor, sensory, behavioral, cognitive, and mood-related functions. Its activity extensively modulates cortical excitability, both in physiological conditions (i.e., sleep-wake cycle and arousal) and in disease (i.e., epilepsies). Such a wide variety of effects arises from the long course and profuse axonal branching of isodendritic reticular neurons, which allows the neuronal message to travel toward the entire cerebral cortex and downstream to the spinal cord. On the other hand, the isodendritic architecture featuring a monoplanar branching allows most RF neurons to cover roughly half of the brainstem and to be impinged by ascending and descending pathways. In parallel, such a generalized influence on CNS activity occurs in combination with highly focused tasks, such as those involved in the coordination of gaze.

Thus, this special issue necessarily encompasses such a multi-faceted nature of the RF. In fact, the integration of multiple activities within the brainstem reticular circuitries may explain why alterations of each of these domains may affect the emotional sphere, paving the way to the concept of emotional brainstem (Venkatraman et al.). This brainstem region was explored in pioneer electrophysiological studies carried out by Moruzzi and Magoun (1949), who first demonstrated a crucial role of this wide area in activating and deactivating cortical EEG background amplitude and frequency. Interestingly, they demonstrated that there is a direct diffuse connection of different levels of RF (ranging from medulla to midbrain) with the whole cortex. At that time, however, the anatomical substrates responsible for such effects were largely ignored, and even the systematic definition of the RF as a complex of specific nuclei was still to be defined. Moreover, also the neurochemical substrates responsible for such effects were still to be discovered. In the following decades the main neurons constituting different areas of RF; and their neuro- and co-transmitters mediators have been characterized. Nevertheless, some biochemical and neuroanatomical features of specific RF neurons still need to be better defined, in different species, including humans. Therefore, a contribution of the present issue is entirely dedicated to a systematic analysis of all catecholamine-containing nuclei within the mouse RF (Bucci et al.). This paper, while confirming classic morphological studies on the isodendritic core of the RF (Brodal, 1957; Ramón-Moliner and Nauta, 1966), sheds new light on a few previously undefined reticular neurons. In fact this study showed that some neurons located in the area postrema are indeed catecholamine cells, placed continuously and downstream to the A2 area (Area Cinerea).

The high connectivity of reticular nuclei may explain why a variety of different sensory information (i.e., visceral, trigeminal, and vestibular) may impact cognitive functions through ascending reticular neurons, pertaining to the catecholamine nucleus Locus Coeruleus (LC) (De Cicco et al.). Consistently, this issue includes an original investigation on how proprioceptive trigeminal afferents may affect attention and arousal via a tight neuroanatomical interaction between the proprioceptive trigeminal mesencephalic nucleus and the LC (Tramonti Fantozzi et al.). The specific role of LC in sustaining cognitive functions is substantiated by its diffuse branching (Brodal, 1957, 1981) and noradrenaline volume transmission (Fuxe et al., 1988, 2015; Agnati et al., 1995; Agnati and Fuxe, 2000) which produces widespread extrasynaptic paracrine effects. In this way LC, apart from a monosynaptic influence on cortical neurons, may affect the neurovascular unit as well (Giorgi et al.; Petit and Magistretti, 2016; Iadecola, 2017). It is well knows that LC activity exerts a powerful modulation of astrocytes, pericytes and microglia (Heneka et al., 2010; O'Donnell et al., 2012; Iravani et al., 2014). These extraneuronal effects might explain the role of microglial phagocytosis in sleep disorders (Nadjar et al.). Glial cells are also critical for releasing cytokines and chemokines messengers with both proinflammatory and neuroprotective actions. This may lead to an endogenous neuroprotective effect mediated by P27R receptors, as demonstrated by Lim et al.

Within this framework, Giorgi et al. stress the role of LC in modulating the neurovascular unit as a possible mechanism counteracting neurodegeneration in Alzheimer's Disease. This may add on novel cell-to-cell-based pathogenic effects in which misfolded proteins may spread monosynaptically from reticular axons to cortical neurons, according to a prion-like pattern (Giorgi et al.).

For instance, specific patterns of neuronal loss affecting catecholamine-containing reticular nuclei may produce a constellation of phenotypes in Parkinson Disease (PD). In fact, depending on which reticular nucleus is affected, a variety of both motor and non-motor (autonomic, sleep and mood-related, behavioral, and cognitive) symptoms, may occur. This mostly applies to non-motor symptoms, which appear to underlie different PD subtypes, each one owing a specific pattern of brainstem involvement (Gambardella et al.). Frequently, the onset of PD, instead of consisting of motor disturbances, coincides with autonomic alterations and pain. In this regard, the role of the RF in driving painful stimuli, and controlling pain-related circuitries, was reviewed by Martins and Tavares. These authors centered brainstem pain control in a reticular loop, which includes the periaqueductal gray, the rostro-ventro-medial medulla and the ventro-lateral medulla (Martins and Tavares).

The key role of the brainstem RF in mediating those activities relevant to species survival, such as pain and reward, sets the ground for these brain regions as preferential targets for drugs of abuse as reported by Ferrucci et al. In particular, while most of the literature on the effects of amphetamines has focused on their effects on dopaminergic neurons, there are several reports indicating a key role of the effects of amphetamines on LC in mediating many of their behavioral effects, including reward. Furthermore, interesting data indicate that the interaction of RF pontine cholinergic neurons (Ch5 and Ch6) with midbrain DA neurons might be crucial for the hyperlocomotion induced by amphetamines (Ferrucci et al.).

So far, the RF has been viewed mainly as an archaic collection of ascending and descending systems, and interconnected nuclei, which play only a rough and ancestral role in interlacing various CNS areas. Nevertheless, specific nuclei of the RF act as premotor centers, involved in the fine-tuning of the gaze, both along the vertical and horizontal plane. This latter function was investigated by Wang et al. who defined the central mesencephalic reticular formation as a conduit for the collicular saccadic signals in the horizontal gaze (Wang et al.).

All these features are covered by specific contributions of the research topic, which offers an updated view to define the anatomical correlates of the multiple and interconnected roles played by the brainstem reticular formation in health and disease.

## Author Contributions

All authors listed have made a substantial, direct and intellectual contribution to the work, and approved it for publication.

### Conflict of Interest Statement

The authors declare that the research was conducted in the absence of any commercial or financial relationships that could be construed as a potential conflict of interest.
